# Supplementary material for: Mechanistic Studies for Palladium Catalyzed Copolymerization of Ethylene with Vinyl Ethers
Source: Polymers (Basel). 2020 Oct 19;12(10):2401. doi: 10.3390/polym12102401 (PMC7603233; doi:10.3390/polym12102401)
Supplement: Supplementary file 1 [file polymers-12-02401-s001.pdf]

polymers

Article

Mechanistic studies for palladium catalyzed copolymerization of ethylene with vinyl ethers

Andleeb Mehmood [1,†], Xiaowei Xu [1,†], Waseem Raza [1], Ki-Hyun Kim [2,*] and Yi Luo [1,*]

[1] State Key Laboratory of Fine Chemicals, School of Chemical Engineering, Dalian University of Technology, Dalian 116024, China; andleeb.mehmood@gmail.com (A.M.); xuxiaowei001@mail.dlut.edu.cn (X.X.); razawaseem2@yahoo.com (W.R.)

[2] Department of Civil and Environmental Engineering, Hanyang University, 222 Wangsimni-Ro, Seoul 04763, Korea

* Correspondence: kkim61@hanyang.ac.kr (K.-H.K.); luoyi@dlut.edu.cn (Y.L.)

† Two authors contributed equally

Received: 9 October 2020; Accepted: 16 October 2020; Published: 19 October 2020

Supporting Information

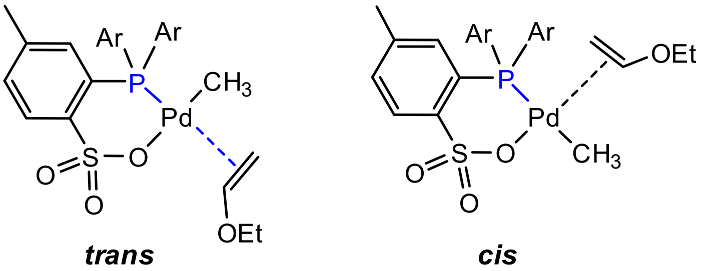

Figure S1. Two coordination manners (*trans* and *cis*, Ar = 2-MeOC$_6$H$_4$) of the VE corresponding to P-atom of phosphine-sulfonate based Pd complexes.

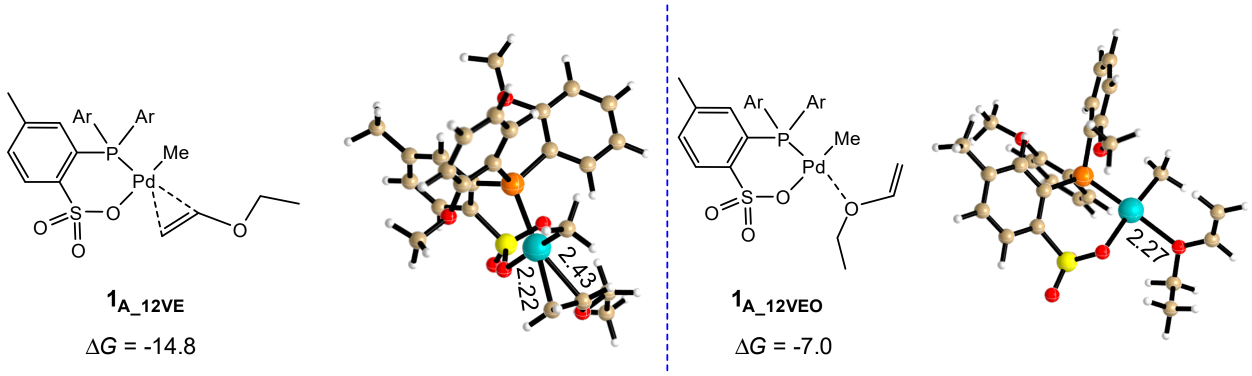

Figure S2. Coordination of the VE as **1**$_{A\_12VE}$ and **1**$_{A\_12VEO}$.

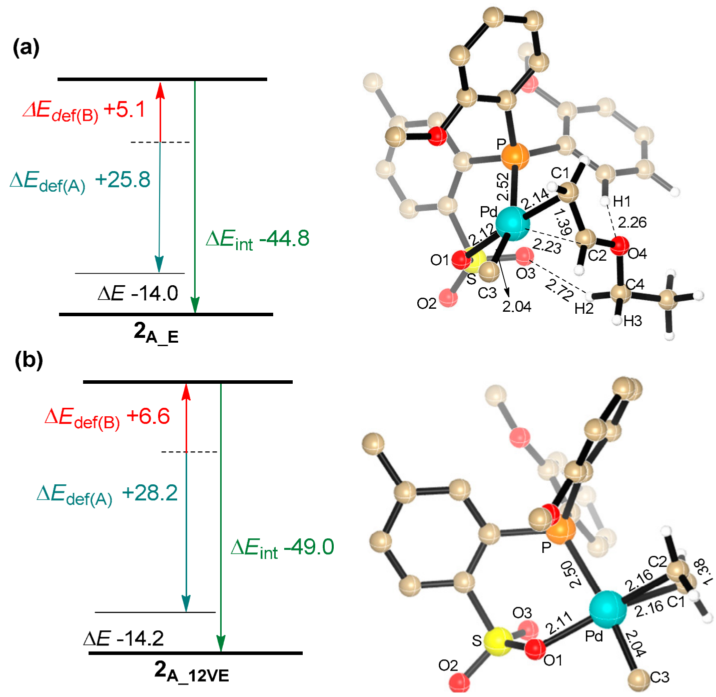

Figure S3. Distortion/interaction for the π-complexes: (a) **2**$_{A\_E}$ and (b) **2**$_{A\_12VE}$.

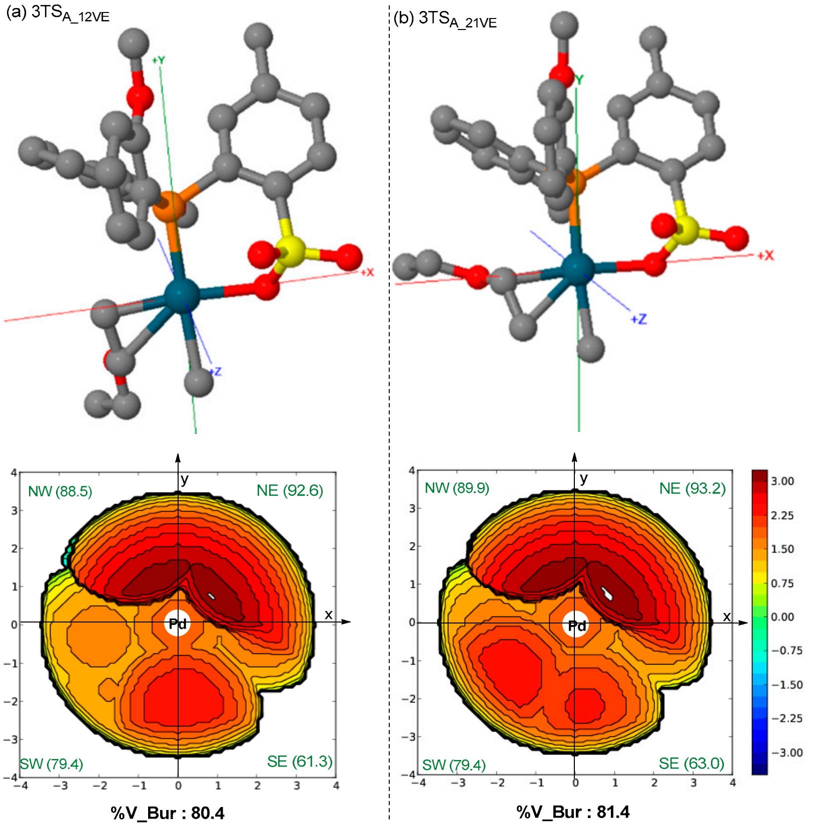

Figure S4. Steric maps for TSs. (a) **3TS**$_{A\_12VE}$, (b) **3TS**$_{A\_21VE}$.

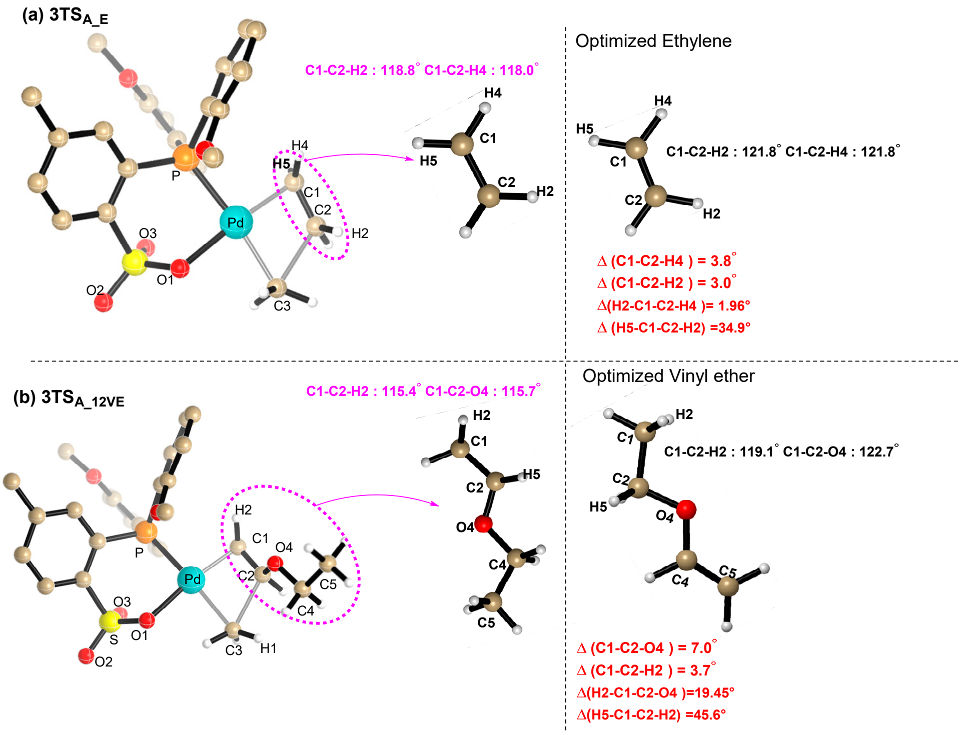

Figure S5. Comparison between geometric parameters for ethylene and vinyl ether insertion of TSs. (a) **3TS**$_{A\_E}$, (b) **3TS**$_{A\_12VE}$ and optimized ethylene and vinyl ether.

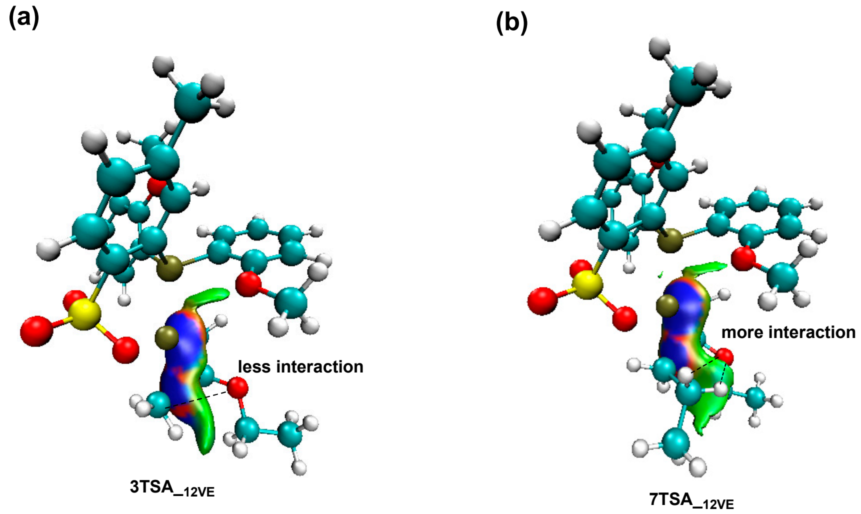

Figure S6. Noncovalent interaction analysis (NCI) for transition states: (a) **3TS**$_{A\_12VE}$ and (b) **7TS**$_{A\_12VE}$.

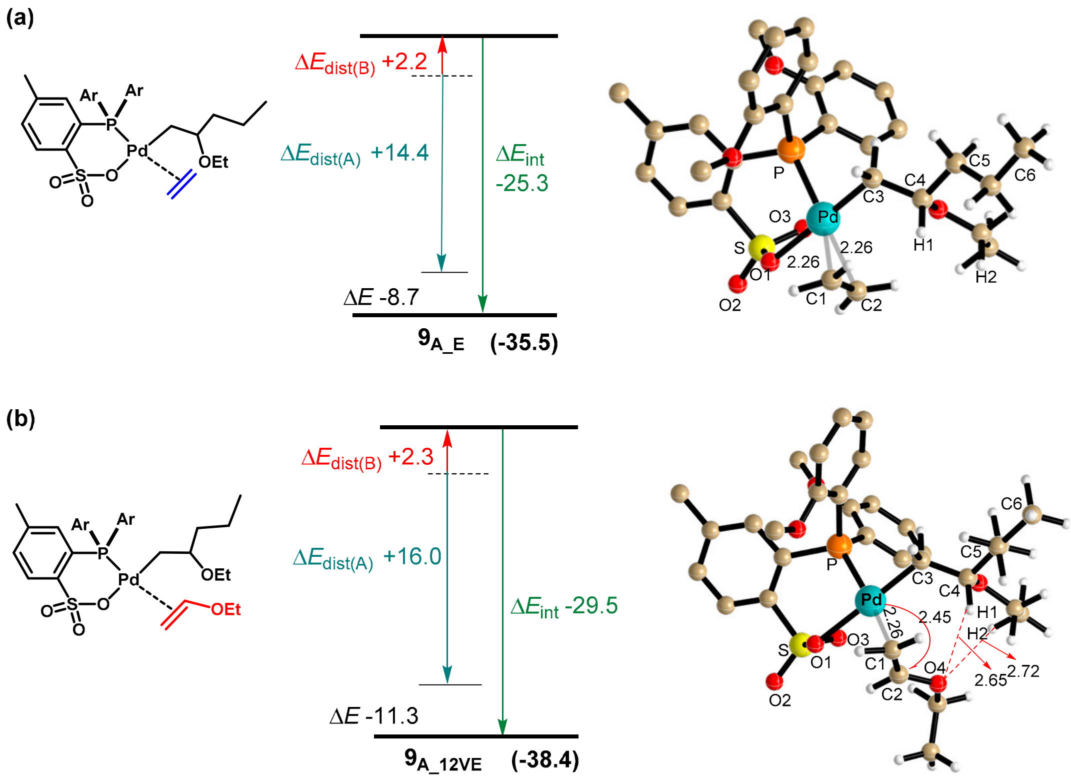

Figure S7. Distortion/interaction for the π-complexes: (a) **9**$_{A\_E}$ and (b) **9**$_{A\_12VE}$.

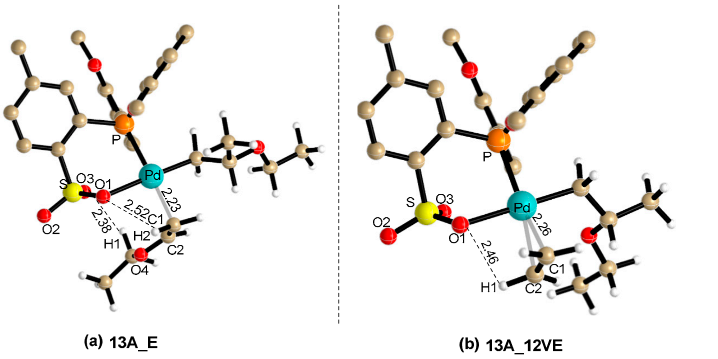

Figure S8. Interaction for the π-complexes: (a) **13**$_{A\_E}$ and (b) **13**$_{A\_12VE}$.

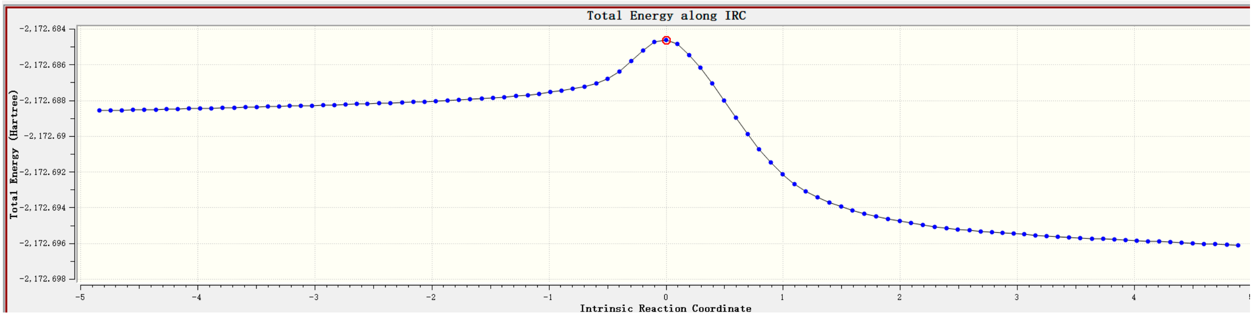

Figure S9. IRC plot.

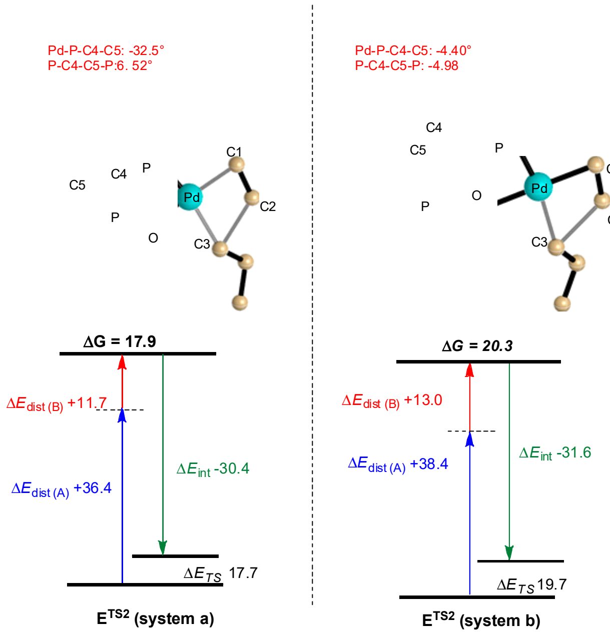

Figure S10. Distortion/interaction analysis of the ethylene homo polymer formation for second ethylene insertion by catalyst systems "**a**" and "**b**" along with dihedral angles (in deg) and bond lengths (in Å) demonstrated in structures.

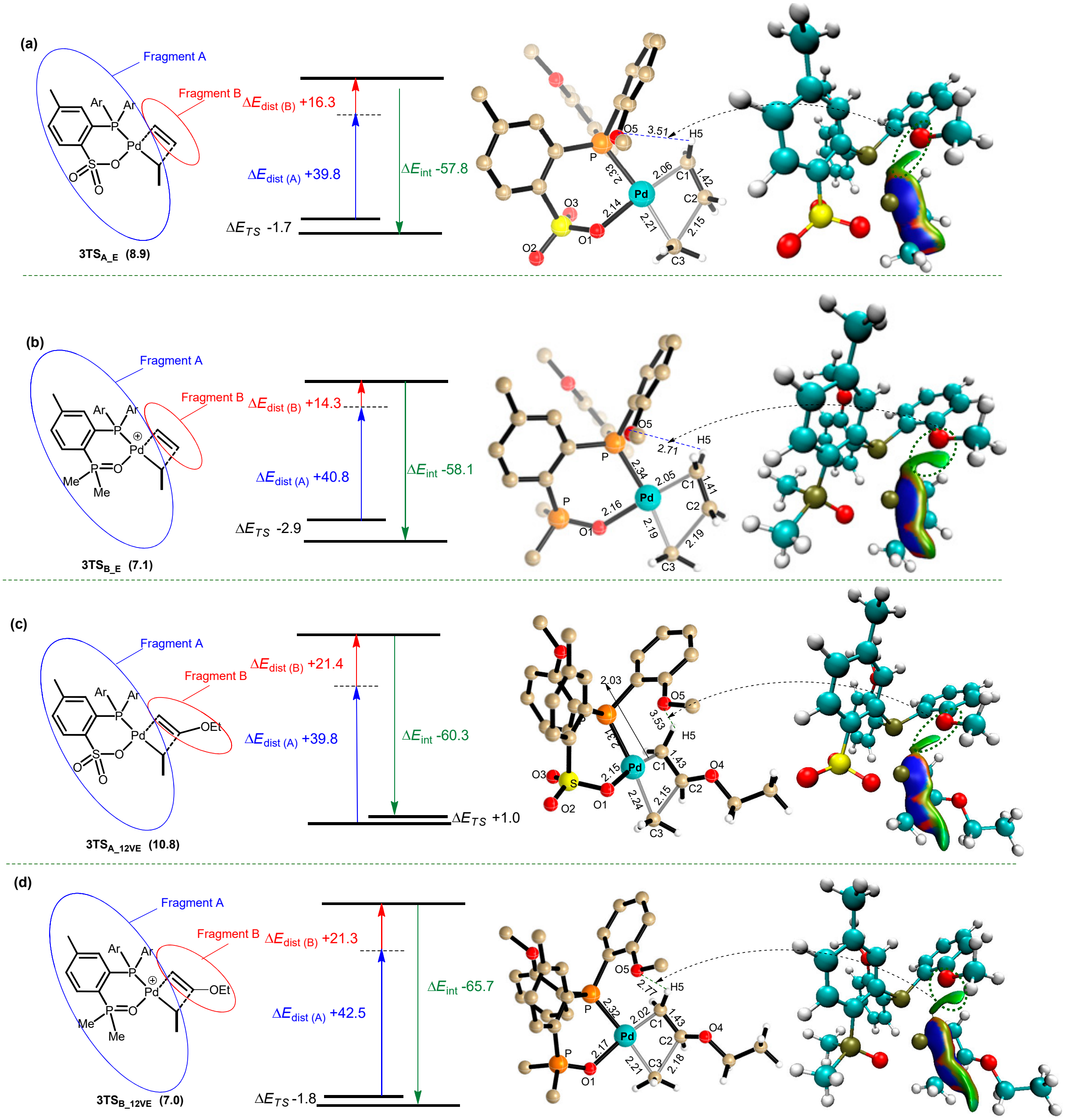

Figure S11. Distortion/interaction analysis of the transition state **3TS**$_{A\_E}$ (a), **3TS**$_{B\_E}$ (b), **3TS**$_{A\_12VE}$ (c), and **3TS**$_{B\_12VE}$ (d). Energy in kcal/mol and distances in Å. Hydrogen atoms of the catalyst's ligand have been omitted for clarity.

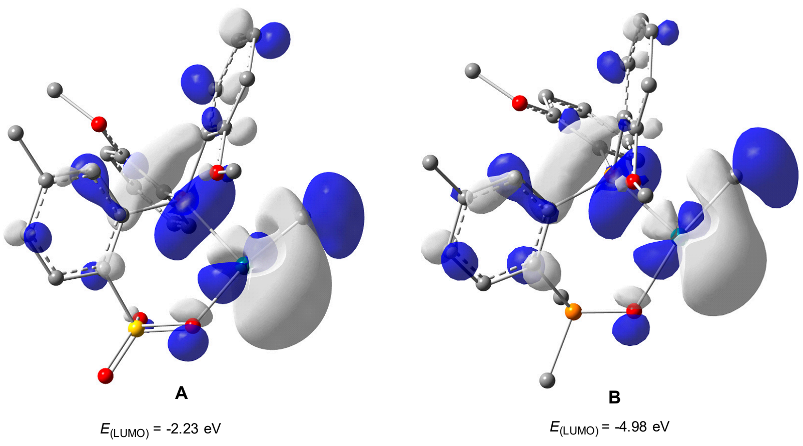

Figure S12. LUMO plots and energies of the two catalysts (**A** and **B**).

© 2020 by the authors. Submitted for possible open access publication under the terms and conditions of the Creative Commons Attribution (CC BY) license (http://creativecommons.org/licenses/by/4.0/).
